# Recent Advances in Preventative Medicine*Abstract of the Address in State Medicine, delivered before the American Medical Association, at the Thirty-third Annual Meeting held at Chicago, Ill., June 7–10, 1887.

**Published:** 1887-08

**Authors:** Geo. H Rohe

**Affiliations:** Baltimore, Md.


### Recent Advances in Preventative Medicine.*

BY GEO. H ROHE, M.D., OF BALTIMORE, MD.

(Professor of Hygiene in the College of Physicians and Surgeons.)

Progress in any branch of science or art may be measured either by the number and character of the new discoveries made, or by the gradual advances in the application of knowledge previously acquired. Judged by either of these criteria, the record for State Medicine during the past year is a creditable one.

In the field of epidemiology and endemiology, the progressive extension of the fifth great pandemic of cholera first claims attention. Extinguished in the portions of Italy, France, and

* Abstract of the Address in State Medicine, delivered before the American Medical Association, at the Thirty-third Annual Meeting held at Chicago, Ill., June 7—10, 1887.

Spain, ravaged in 1885 and 1886, it has slowly invaded south-eastern Italy, Hungary, and other Austrian possessions, and has been imported into South America, whence it threatens the United States by several routes. The danger of invasion of this country is at present greater than at any time within the past three years.

Yellow fever inoculation, as practiced by Friere in Brazil, and Carmona in Mexico, has claimed a large share of the attention of sanitarians during the year. The claims made in favor of this method of preventing this scourge are now being subjected to an official investigation authorized by the United States government.

Diligent search has been made for the specific organism supposed to be the infective agent in vaccine virus, but without definite success. The results obtained are not entirely negative, however, and one may cherish the hope that a solution of this problem will soon be reached.

The relation of a peculiar disease of cows to scarlet fever, and the discovery of a specific microbe in the blood in the latter disease, have attracted much attention. The restriction of scarlet fever will doubtless be more thoroughly effected as soon as physicians are convinced of its bacterial nature, and clearly comprehend its mode of transmission. Statistics are given showing what has already been accomplished in this field.

Sternberg, Frankel, and Weichselbaum have studied the specific microbe of croupous pneumonia, which the former regards as identical with his *micrococcus Pasteuri*; in which opinion both the other authors mentioned coincide. Dr. Baker, of Michigan, has also shown that croupous pneumonia seems to be dependent upon a cold, dry atmosphere.

Measures for the restriction of pulmonary tuberculosis are adverted to. Tuberculous patients should not be treated in the same hospital wards with non-tuberculous individuals, and prompt disinfection of the sputa and other discharges should be practiced, in order to diminish opportunities for infection. General sanitary measures should, however, not be neglected in the warfare upon the bacillus. There is danger that a too exclusive attention to the microbian factors of disease will narrow our views of epidemiology and preventive medicine.

It seems to be established that the micro-organism discovered in the intestinal lesions and discharges in typhoid fever is the caues of this disease. The fact that this microbe may preserve its vitality for a considerable time in water and ice has been shown by Bolton, Wolfthugel, Prudden, and others. This, together with the well-known history of outbreaks of this disease undoubtedly depending upon pollution of drinking water, should make prompt measures of disinfection imperative in every case. The physician fails in his duty who neglects measures for the thorough destruction of the typhoid infection existing in the intestinal discharges.

The importance of disinfection of bedding, clothing, and other personal and household articles in contagious diseases, demands that health authorities should have under their control establishments where disinfection can be carried out on a large scale and at public expense. Such institutions are now in use in Berlin, Dusseldorf, Gottingen, Strasburg, Breslau, Leipzig, Danzig, and other cities in Europe. The results are pronounced to be exceedingly beneficial. Steam under pressure is regarded as the best disinfecting agent.

Quarantine, a word which for more than five centuries has been synonymous with barbarism, is becoming under modern methods a safeguard to the public against infection, and an advantage instead of obstruction to commerce. The results achieved at the model quarentine station at New Orleans encourage the hope, and almost warrant the prediction, that the days of the quarentine of detention, whether by sea or land, are past, and that quarentine in future will mean simply *thorough disinfection* of formites, and, of course, effective isolation of persons already infected.

Cremation of garbage seems to be the best method yet devised for the inoffensive destruction or final disposal of solid city wastes.

The irrigation system of sewage disposal has steadily won favor. In Berlin, Breslau, and Danzig, in Germany, Birmingham in England, and Pullman and other places in this country, it has been in successful operation. Chemical precipitation and purification of sewage has also been adopted with satisfactory results in various German cities. A board of distinguished engineers recently recommended the same system for the city of Providence, Rhode Island.

Professor Vaughan's discovery of a very poisonous ptomaine in cheese, ice cream, and milk, undergoing certain chemical changes, has been confirmed by a number of investigators in various parts of the country. Vaughan's suggestion that tyrotoxicon may be found to be the poison which produces cholera infantum, opens up a new field for investigation in which every physician must of necessity be interested.

Analyses of food and drugs made during the year in Massachusetts and New York, show the wide extent to which adulteration is practiced, and how the people are defrauded. Among the most startling instances are olive oil, of which 68 samples out of 91 were spurious. Vinegar was adulterated in 79 samples out of 116; mustard 124 times in 211; white pepper 63 times in 128; black pepper 41 times in 71; mace 29 times in 45. Of 9 samples of horseradish examined, only 1 was found genuine. A precipitate of uncrystallizable sugar and coloring matter and chloride of tin (poisonous) is sold to candy makers for making confectionery. Citrate of quinine and iron from respectable manufacturers contain 3½ per cent. of quinine, instead of 12 per cent. demanded by the pharmacopœia. Authority and means should be given to the health authorities to protect the public from these frauds, many of which are sources of danger to life and health.

Statistics collected by the speaker show that five-sixths of the inhabitants of cities in this country have no facilities for bathing, except such as are afforded by a pail and sponge, or an easily accessible river, lake, or other body of water. The establishment of public baths is urgently recommended both as a sanitary as well as moral measure. Tub or pool baths are objectionable, both on account of expense and lack of privacy in the latter. The spray baths in use in the German and French army barracks are recommended. These are not expensive, either in the first cost or administration, and allow each bather absolute privacy and the opportunity for a thorough cleansing in clean water. Public baths should be opened the year round, and not only during the summer.

A number of instances are grouped together showing how the enforcement of appropriate sanitary measures has saved life. In Michigan the saving of life from one disease (scarlet fever) has amounted during the last eleven years to 3,718, or 338 per year. In 1886, appropriate sanitary measures saved the lives of 298 persons who would have died of diphtheria if such measures had not been enforced. In England and Wales, the annual average saving of life due to sanitary measures has amounted in the five years ending 1885, to 62,000. In Baltimore, a marked reduction of deaths from infectious diseases has followed the enforcement of certain sanitary precautions. In Memphis the death rate has been reduced in six years from 35 per thousand to 23.80 per thousand. In Chicago the reduction in mortality in the last five years has been from 25.69 per thousand to 19 46 per thousand, a net saving of 17,214 lives in that city during that period.

While all advances in sanitary administration have doubtless contributed to produce these good results, the main influence is to be attributed to three factors. These are *compulsory notification of infectious diseases; prompt and effective isolation of the sick and infected, and thorough disinfection of all infected articles aud sources of infection.* These must be the watchwords of the practical sanitarian of the future.

